# Infiltrative solid papillary carcinoma of the breast with axillary lymph node metastasis: a case report

**DOI:** 10.1186/s12905-023-02596-6

**Published:** 2023-08-28

**Authors:** Xiaowei Zhang, Bifei Huang, Kangbin Wu, Saiping Fu

**Affiliations:** 1 Department of Pathology, Affiliated Dongyang Hospital of Wenzhou Medical University, Dongyang, 322100 Zhejiang P. R. China; 2 Department of Breast Disease Diagnosis and Treatment Center, Affiliated Dongyang Hospital of Wenzhou Medical University, Dongyang, 322100 Zhejiang P. R. China

**Keywords:** Aggressive growth, Breast, Metastasis, Solid papillary carcinoma, Surgery

## Abstract

**Background:**

Solid papillary carcinoma (SPC) is a rare breast papillary tumor variant. The main histological features of SPC consist of neoplastic cell-rich nodules with thin fibrovascular cores, often accompanied by mucous secretion and neuroendocrine differentiation. Infiltrative solid papillary carcinoma (ISPC) tumor cells have an invasive, map-like growth pattern with serrated irregular growth. Due to its unique clinicopathological features, SPC is classified as two pathological tissue types based on the 2019 WHO classification of breast tumors: SPC in situ and ISPC.

**Case presentation:**

We report a case of a 55-year-old female patient who was admitted to the hospital due to a painless left breast mass that had persisted for two years. Mammography suggested a mass in the left upper outer quadrant (BI-RADS 4B), and ultrasound of the breast demonstrated a cystic mass of the left breast (US_BI_RADS 4 C) with multiple enlarged lymph nodes in the left axilla. Postoperative pathology revealed ISPC with one lymph node metastasis in the left breast. Modified radical mastectomy was performed on the left breast. Subsequently, the patient received letrozole endocrine therapy, epirubicin hydrochloride and cyclophosphamide chemotherapy, and radiotherapy of the left chest wall and left upper and lower clavicular regions. After 17 months of follow-up, there was no evidence of recurrence or distant metastasis.

**Conclusions:**

SPC is a group of heterogeneous tumors. SPC in situ has a good prognosis. In contrast, ISPC has a unique histological morphology and growth pattern with invasive biological behavior that can lead to lymph node and distant metastases.

## Background

Solid papillary carcinoma (SPC), a type of breast papillary carcinoma, is predominantly found in older female patients and has unique clinicopathological features [1]. Infiltrative solid papillary carcinoma (ISPC) is a newly reported distinct type of SPC with an invasive growth pattern and specific clinicopathological features. This study reports a case of ISPC with lymph node metastasis to explore its clinicopathological features and provide evidence for its differential diagnosis.

## Case description

A 55-year-old female patient was admitted to the hospital on May 23, 2021 for a left breast mass that had persisted for 2 years. The patient reported noticing a painless mass in the left breast approximately 2 years prior when it was the size of a peanut. The mass gradually increased to the size of a quail egg. Surgical excision was recommended.

Upon physical examination, the patient had no nipple depression or discharge. A 3-cm diameter mass was palpable in the left breast and was solid, mobile, well bounded, and without tenderness. There was no significant lymph node enlargement in either axilla.

**Fig. 1:**
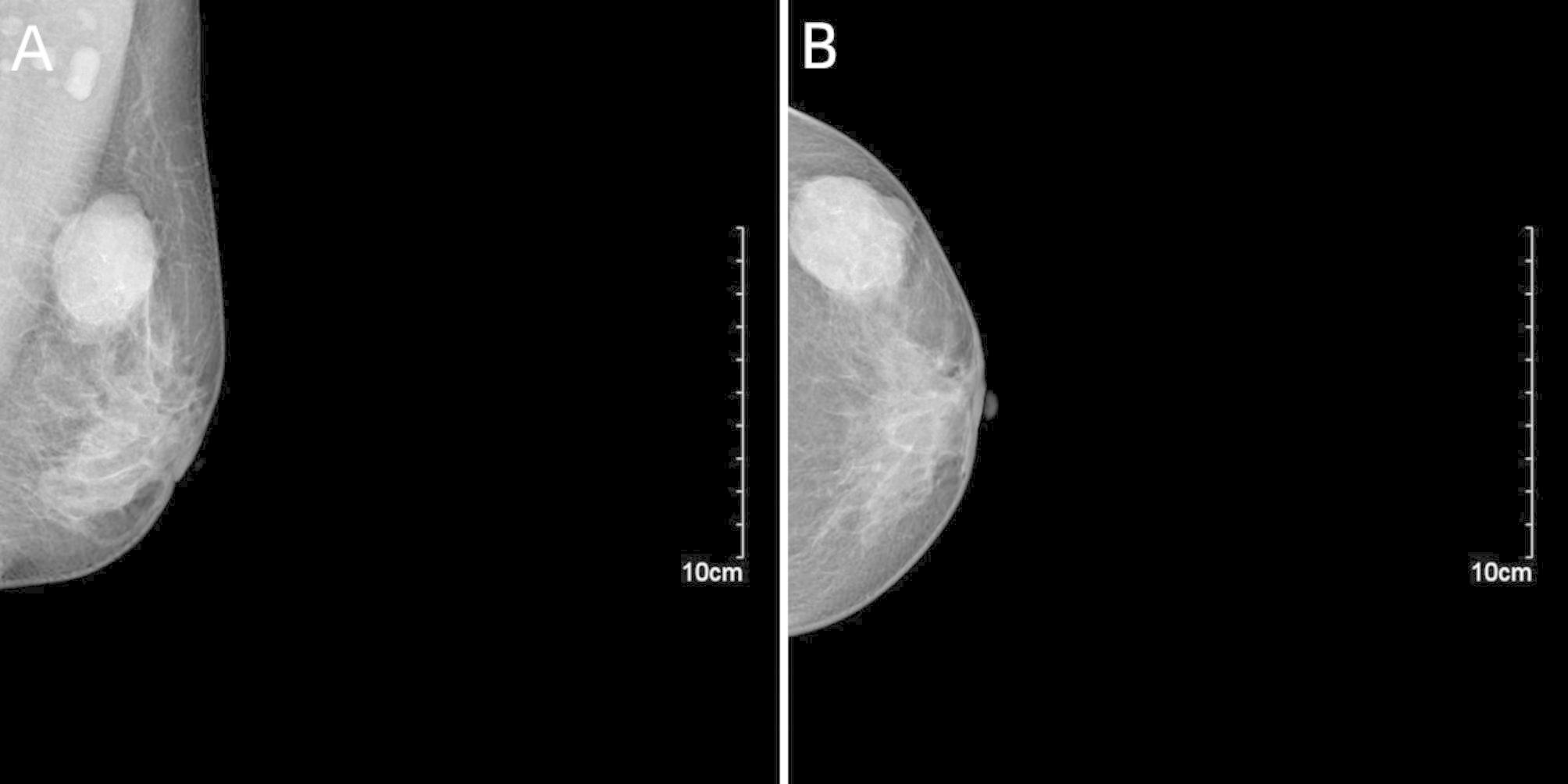
Mammography of this case. There is a high-density mass in the upper outer quadrant of the left breast with clear boundaries and no abnormal calcification (A, B)

Mammography revealed a 4.0 cm × 3.2 cm × 4.0 cm high-density mass with clear boundaries in the upper quadrant of the left breast. No abnormal calcification was observed in the left breast. However, several lymph nodes were visible in both axillae, with one particularly enlarged in the left axilla. The mammography diagnosis, using a molybdenum target, revealed a left upper quadrant external breast mass (BI-RADS 4B) with bilateral enlarged axillary lymph nodes, particularly one in the left axilla (Fig. 1).

**Fig. 2:**
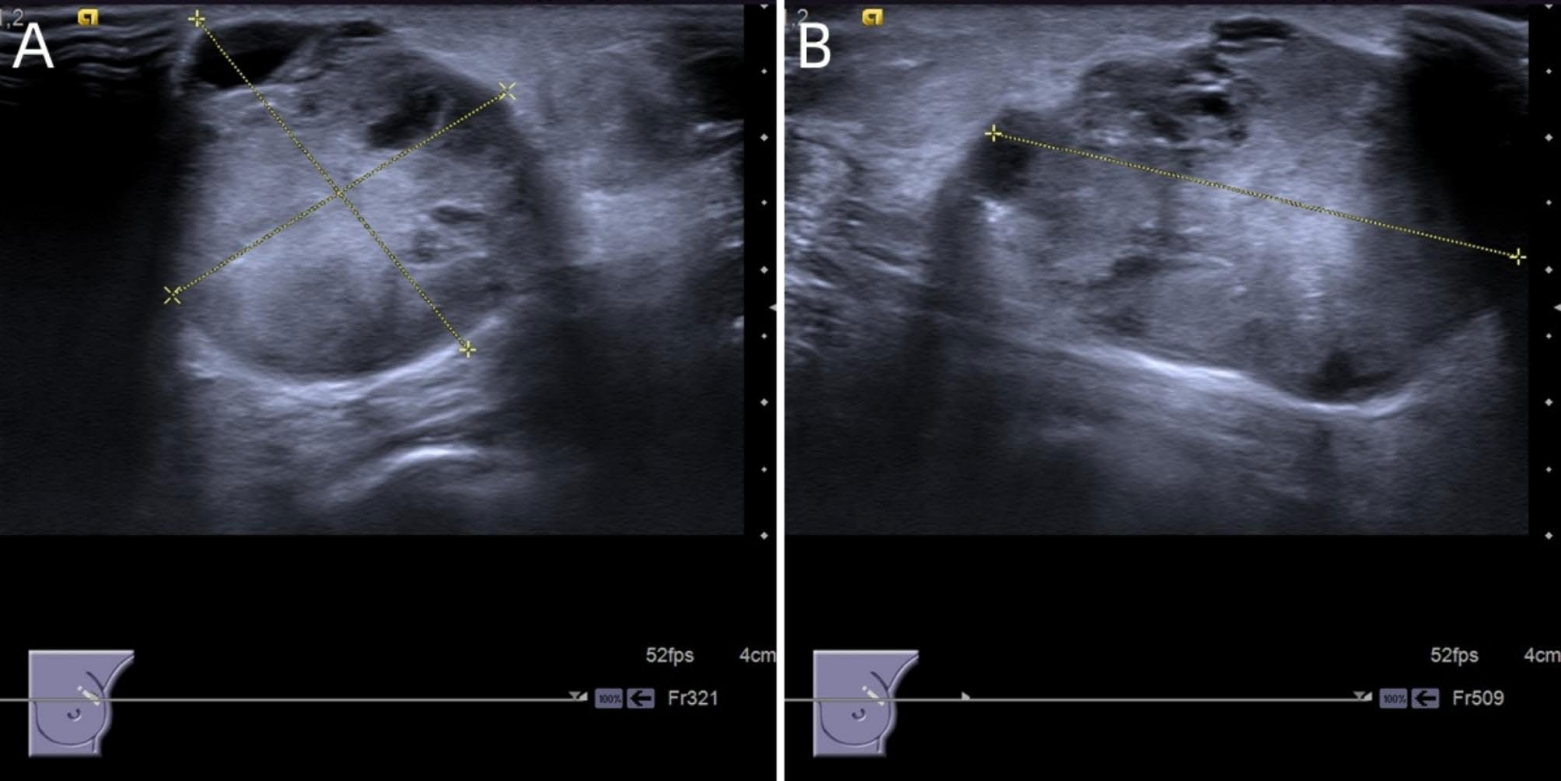
Ultrasound findings of this case. In the left breast, there is an elliptic mixed echoic mass dominated by a solid hypoechoic mass with smooth edges (A, B)

Breast ultrasound identified a 41 mm × 32 mm × 30 mm mass at the 1 o’clock position of the left breast. This was noted to be growing horizontally and was mostly oval. The edge of the mass was smooth, and the interior was a cystic solid mixed echo pattern dominated by solid hypoechoic mass, with multiple gritty strong echoes visualized inside the mass. An enhanced echo was also noted behind the mass. Color Doppler flow imaging showed a mixed and abundant blood supply. Multiple lymph nodes of different sizes could be seen in the left axilla. The largest one was 10 mm × 7 mm in size, oval, and with a clear boundary and uniform internal echo. The structure of the lymphatic hilum was not clearly visualized, and the boundary between the cortex and medulla was not clear. The ultrasonic diagnosis was, therefore, a cystic mass of the left breast, US_BI_RADS 4 C, with multiple enlarged lymph nodes in the left axilla (Fig. 2).

After admission, the relevant preoperative examinations were completed, including blood routine test, liver and kidney function, coagulation function, breast ultrasound, and lung computed tomography examination. The patient underwent segmental mastectomy of the left breast under local anesthesia. Prior to resection of the left breast mass, a rapid frozen section was sent for pathological examination; the report was invasive carcinoma of the left breast. Subsequently, modified radical mastectomy was performed for the left breast. Intraoperatively, the 4.0-cm mass was firm, with clear boundaries, and the left axillary lymph nodes were enlarged. The procedure went smoothly with intraoperative bleeding of approximately 10 mL.

**Fig. 3:**
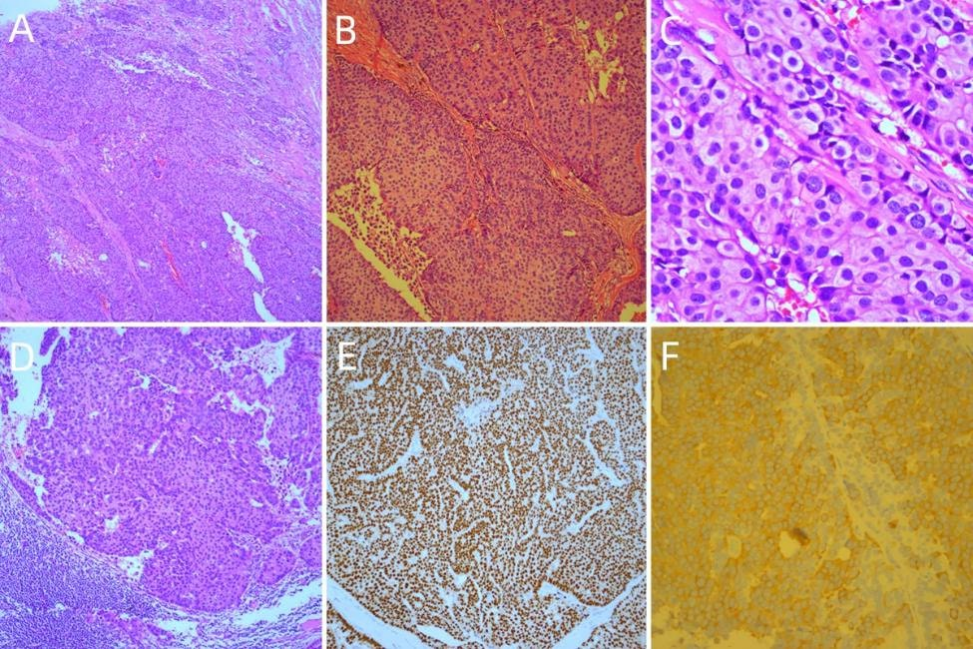
Histologic findings of this case. The tumor cell nest displays irregular infiltrating growth, with solid cell nests and thin fibrovascular cores. The tumor cells are oval with endocrine characteristics, eosinophilic granular cytoplasm, and delicate nuclear chromatin (A, ×50; B, ×100; and C, ×400). Nest metastases of tumor cells are found in axillary lymph node (D, ×100). (E) Tumor cells strongly express estrogen receptors (ER) (Immunohistochemistry, × 100). (F) Tumor cell nests strongly express neuroendocrine markers for synaptophysin (SYN) (Immunohistochemistry, ×200)

Postoperative microscopy confirmed that the tumor tissue was solid and nodular, with a fibrovascular core, and that some cellular nests were irregular, displaying serrated or cartographic infiltrative growth. ISPC tumor cells were small to medium in size, with round or ovoid morphology. Part of the cytoplasm was eosinophilic or displayed light granular staining, and mitosis was rarely visualized. The pathological diagnosis was ISPC (4.5 cm × 2.8 cm), with no obvious vascular invasion and nerve involvement. Of the 21 axillary lymph nodes examined, one showed cancer metastasis and lymph node invasion (metastasis size 0.6 cm × 0.5 cm). Nipple, skin, and basal margin were negative for malignant infiltration (Fig. 3A-D).

Immunohistochemical staining results indicated that ISPC tumor cells were strongly positive for estrogen receptor (ER) (80%) and progesterone receptor (PR) (70%). ISPC tumor cells were also positive for synaptophysin (SYN) and E-cadherin. The specimen was negative for human epidermal growth factor receptor-2 (HER2), chromogranin A (CgA), and CD56; myoepithelial cells were negative for p63 and calponin. The Ki-67 index was 20% (Fig. 3E, F).

Postoperatively, the patient’s pathological stage was determined to be PT2N1M0, with a clinical stage of IIB. Postoperative letrozole endocrine therapy, epirubicin hydrochloride, and cyclophosphamide chemotherapy, and left chest wall and left upper and lower clavicle radiotherapy, were initiated. After 17 months of follow-up, no recurrence or distant metastasis was observed.

## Discussion and conclusions

Papillary lesions of the breast are a heterogeneous group of benign and malignant tumors occurring primarily in the central region of the breast. Commonly used to describe both in situ and invasive breast cancer, papillary carcinoma is nonspecific in indicating overlapping histological features and clinical, biological behavior [1, 2]. SPC of the breast is a rare tissue subtype of breast papillary lesions, accounting for less than 1% of all breast cancers [3]. ISPC, an invasive breast cancer with unique clinicopathologic features, is classified as an independent subtype by the 2019 WHO Classification of Breast Tumors [4]. Reports on this type of carcinoma are scarce, and its clinical symptoms are similar to those of SPCs in situ. ISPC occurs primarily in patients aged between 60 and 80 years [4]. Most patients with ISPC find a painless breast mass or present with abnormal imaging findings, and some patients experience nipple discharge and blood discharge.

In the present case, pathological examination of the ISPC revealed a solid grayish-brown or grayish-yellow nodular mass with soft texture; no envelope was observed in the general profile. The boundary of the nodules was characteristic of invasive cancer, and more components of invasive cancer caused a fuzzier boundary of the tumor. Microscopic diagnosis of ISPC can create diagnostic confusion and is mainly based on the growth pattern, with myoepithelial cells aiding the diagnosis of SPC [5–7]. If myoepithelium is present around the nested cells, it is generally considered to be SPC in situ. However, the absence of myoepithelial markers does not necessarily indicate expansive tumor invasion. Only when the SPC tumor cell nest has a serrated, irregular border, and myoepithelial cells around the tumor nest (myoepithelial markers P63, Calponin, and SMA negative) are absent, is this presentation considered to be an ISPC. In the tumor, a thin fibrovascular cores can be seen microscopically in the nest of solid cell hyperplasia, which can be arranged in a flow pattern. ISPC tumor cells are oval or spindle, may have endocrine characteristics, and have eosinophilic granular cytoplasm. The nuclear chromatin is delicate, the nucleolus is small and centered, the nuclear grade is mostly mild to moderate, and intracellular and extracellular mucus secretion is common. All tumor components are positive for ER, PR, and express neuroendocrine markers, such as SYN, CgA, CD56, but most are negative for HER2.

ISPC can be distinguished from the following pathological tissue type: (1) SPC in situ (both types are determined by microscopic morphology). If the cancer nest has a regular shape, neat edges, expansion and growth, and no evidence of invasion, regardless of the presence of myoepithelial cells, it will be classified as carcinoma in situ. ISPC is diagnosed if the nest is unorganized, with serrated or map-like changes, a more pronounced fibrous interstitial reaction around the nest, and complete myoepithelial disappearance. (2) Encapsulated papillary carcinoma of the breast is a key distinguishing feature of this subtype in that it is mostly a solitary cystic nodular lesion, surrounded by thick encapsulated fibrocystic walls and does not express endocrine markers by immunohistochemistry. (3) Invasive papillary carcinoma of the breast occurs when tumor tissue shows papilla-like growth patterns, with complex branching papillae with fibrovascular cores at the center of the papillae. Invasive papillary carcinoma tumor cells are columnar, the cytoplasm is eosinophilic with light staining, and neuroendocrine markers are not expressed by immunohistochemistry, which makes them an easy subtype to distinguish from ISPC. (4) Coventional type intraductal papilloma is generally lacking a thin fibrovascular cores, with negative neuroendocrine markers and positive myoepithelial markers.(5)Lobular carcinoma lacks a fibrovascular cores and cell adhesion, and does not express E-cadherin. (6) Neuroendocrine carcinoma is typically characterized by a lack of mucus and a papillary structure with a thin fibrovascular cores, but neuroendocrine markers are also strong expressed. (7) Invasive ductal carcinoma, lacking a papillary structure with fibrovascular cores, generally does not express neuroendocrine markers.

ISPC has an aggressive growth pattern and may metastasize, but the prognosis is usually better than that of nonspecific invasive carcinomas [8–10]. Surgical treatment is the first choice for the management of ISPC. Comprehensive postoperative treatment is selected according to the clinical stage and molecular classification of the tumor itself. In our case, the patient underwent a modified radical mastectomy for breast cancer. Due to the presence of lymph node metastasis, the clinical stage was stage IIB, and postoperative endocrine and chemoradiotherapy were combined and administered to the patient as adjuvant therapies.

We report a case of ISPC with axillary lymph node metastasis. ISPC has unique features in both its clinical manifestations and histopathology. It is a unique type of breast invasive carcinoma. The biological behavior of SPC is invasive, with lymph node or distant metastasis. However, due to the limited number of reported cases, further research is needed for diagnosis and treatment.

## Data Availability

Not applicable.

## Abbreviations

CgA: chromogranin A
ER: estrogen receptor
HER2: human epidermal growth factor receptor-2
ISPC: infiltrative solid papillary carcinoma
PR: progesterone receptor
SPC: solid papillary carcinoma
SYN: synaptophysin

## References

[1] Kulka J, Madaras L, Floris G, Lax SF (2022). Papillary lesions of the breast. Virchows Arch.

[2] Wei S (2016). Papillary lesions of the breast: an update. Arch Pathol Lab Med.

[3] Şenel F, Karaman H, Eroğlu M, Tuna Ö (2017). Invasive papillary breast carcinoma, solid variant with neuroendocrine differentiation. Turk J Surg.

[4] WHO Classification of Tumours Editorial Board (2019). Breast tumours. WHO classification of tumours.

[5] Lin X, Matsumoto Y, Nakakimura T, Ono K, Umeoka S, Torii M (2020). Invasive solid papillary carcinoma with neuroendocrine differentiation of the breast: a case report and literature review. Surg Case Rep.

[6] Tacchini D, Vassallo L, Butorano MA, Mancini V, Megha T (2016). Solid papillary carcinoma of the nipple: an in situ carcinoma or an expansive growth tumor?. Pathologica.

[7] Tan BY, Thike AA, Ellis IO, Tan PH (2016). Clinicopathologic characteristics of solid papillary carcinoma of the breast. Am J Surg Pathol.

[8] Saremian J, Rosa M (2012). Solid papillary carcinoma of the breast: a pathologically and clinically distinct breast tumor. Arch Pathol Lab Med.

[9] Hashmi AA, Iftikhar SN, Haider R, Haider R, Irfan M, Ali J (2020). Solid papillary carcinoma of breast: clinicopathologic comparison with conventional ductal carcinoma of breast. Cureus.

[10] Coyne JD (2007). Invasive solid papillary breast carcinoma with papillary metastasis. Histopathology.

